# Association between oral microbiome and breast cancer in the east Asian population: A Mendelian randomization and case–control study

**DOI:** 10.1111/1759-7714.15280

**Published:** 2024-03-14

**Authors:** Kexin Feng, Fei Ren, Qingyao Shang, Xin Wang, Xiang Wang

**Affiliations:** ^1^ Department of Breast Surgical Oncology National Cancer Center/National Clinical Research Center for Cancer/Cancer Hospital, Chinese Academy of Medical Sciences and Peking Union Medical College Beijing China

**Keywords:** 16S RNA sequencing, breast cancer, case–control study, Mendelian randomization, oral microbiome

## Abstract

**Background:**

The causal relationship between breast cancer (BC) and the oral microbiome remains unclear. In this case–control study, using two‐sample Mendelian randomization (MR), we thoroughly explored the relationship between the oral microbiome and BC in the East Asian population.

**Methods:**

Genetic summary data related to oral microbiota and BC were collected from genome‐wide association studies involving participants of East Asian descent. MR estimates were generated by conducting various analyses. Sequencing data from a case–control study were used to verify the validity of these findings.

**Results:**

MR analysis revealed that 30 tongue and 37 salivary bacterial species were significantly associated with BC. Interestingly, in both tongue and salivary microbiomes, we observed the causal effect of six genera, namely, *Aggregatibacter*, *Streptococcus*, *Prevotella*, *Haemophilus*, *Lachnospiraceae*, *Oribacterium*, and *Solobacterium*, on BC. Our case–control study findings suggest differences in specific bacteria between patients with BC and healthy controls. Moreover, sequencing data confirmed the MR analysis results, demonstrating that compared with the healthy control group, the BC group had a higher relative abundance of *Pasteurellaceae* and *Streptococcaceae* but a lower relative abundance of *Bacteroidaceae*.

**Conclusions:**

Our MR analysis suggests that the oral microbiome exerts a causative effect on BC risk, supported by the sequencing data of a case–control study. In the future, studies should be undertaken to comprehensively understand the complex interaction mechanisms between the oral microbiota and BC.

## Graphical abstract

This study delves into the association between the oral microbiome, including the microbial communities on the tongue and in saliva, and breast cancer risk. Employing Mendelian randomization to leverage genetic variations as instrumental variables, the research integrates a case–control study for robust validation. This approach provides a nuanced understanding of how oral microbial composition may influence the development of breast cancer, while carefully accounting for confounding factors.

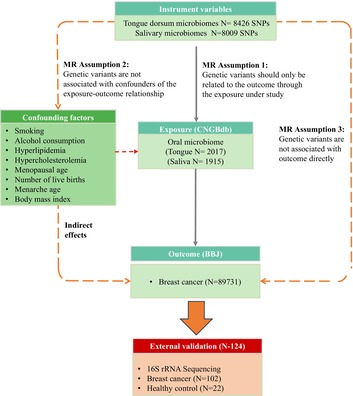

## INTRODUCTION

As an integral part of the human microbiota, the oral microbiome, has been implicated in various pathophysiological processes associated with systemic diseases.^1^ Recently, studies have highlighted the potential association between the oral microbiome and various cancers, including colorectal,^2^ lung,^3^ pancreatic,^4^ and head and neck cancers.^5^

Globally, breast cancer (BC) has the highest incidence and is the second most fatal cancer type. Several risk factors, including hormonal levels, immune system components, genetic predispositions, and lifestyle factors, have been identified for BC. Recently, the microbiome has emerged as a novel and potential contributor to BC, garnering considerable interest within the scientific community. Recent research has highlighted the role of abnormal microbial metabolism in the oral microbiome as a factor contributing to BC development.^6^, ^7^ In particular, it involves the production of toxins that result in DNA damage, alter the metabolism of hormones associated with changes in the microbiome, and affect immune regulation. These mechanisms include the production of extracellular polysaccharides that trigger chronic inflammation, involvement of estrogen metabolism, increasing the levels of hormones associated with BC risk, and production of microbial metabolites that suppress immune responses or promote immune evasion in the tumor microenvironment. Collectively, these findings underscore the importance of additionally investigating the relationship between the oral microbiome and BC, offering novel potential targets for prevention and treatment.^8^, ^9^

However, observational studies in this field are hindered by confounding factors and reverse causality. Therefore, elucidating the potential causal relationships between the oral microbiome and BC is vital for improving their management.

As a versatile strategy, Mendelian randomization (MR) is a method in which whole‐genome sequencing data akin to randomized controlled trials are utilized to explore causal relationships in epidemiology.^10^, ^11^ In MR, genetic variants closely associated with the exposure are utilized as instrumental variables (IVs) to establish causality and alleviate confounding biases. Subsequently, MR presents a robust approach to understand the effect of alterable exposures on the trait of interest, outperforming traditional observational studies.^11^, ^12^

Based on the hypothesis of a reciprocal causal connection between the oral microbiome and BC, in this study, we explored the potential mutual causal association between the oral microbiome and BC via two‐sample MR analysis. Furthermore, we used sequencing data from a case–control study to corroborate this relationship.

## METHODS

### MR study

#### Study design and data sources

The tongue dorsum and salivary microbiome were selected as IVs, and anxiety and breast cancer were used as the outcome variables.

To validate each IV, the single nucleotide polymorphisms (SNPs) utilized in the MR analysis should adhere to three cardinal assumptions^13^: (1) the relevant assumption mandates a potent correlation between the tool and the exposure; (2) the independence assumption necessitates that the SNPs are not associated with any confounding variables that affect the relationship between the exposure and the outcome; and (3) the exclusion restriction assumption dictates that the SNPs affect the outcome solely via the designated exposure, precluding other pathways.

Based on the summary statistics of a recently published genome‐wide association study (GWAS) targeting the East Asian oral microbiome, a two‐sample MR analysis was conducted. Following stringent quality control measures, 2984 individuals (2017 tongue dorsum and 1915 salivary samples) were included. Approximately 10 million common and low‐frequency variants (MAF ≥0.5%) were included. Additional comprehensive information on sample acquisition, sequencing protocols, microbiome trait preparation, and observational and genotyping analyses has been previously described.^14^

The World Health Organization (WHO) defines breast cancer as a disease in which abnormal breast cells grow out of control and form tumors. If left unchecked, the tumors can spread throughout the body and become fatal.^15^ The data for BC were extracted from a comprehensive GWAS involving an Asian population. This dataset comprises 5552 individuals with BC and 89 731 control cases.^16^

#### Selection of genetic IVs

Initially, SNPs were selected at a genome‐wide significance level of P < 5 × 10^−8^. Owing to the absence of oral microbiome‐associated SNPs meeting this stringent benchmark, a moderated significance level of P < 5 × 10^−6^ was selected. A clumping protocol was utilized to neutralize the effects of potential linkage disequilibrium (LD) among the selected SNPs. This entailed setting a radius of 10 000 kb and enforcing an R^2^ cutoff value of <0.001 to extract the SNPs within the LD blocks.^17^, ^18^ To further safeguard allele harmonization, the exposure and outcome datasets were meticulously aligned, removing the SNPs with discordant allele pairings or alleles of intermediate frequency. As a result, a cohort of refined SNPs serving as robust genetic IVs for MR analysis was generated. Furthermore, the F‐statistic of each SNP, individually and cumulatively, was calculated by using the following formula: F = R^2^ × (N − 2)/(1 − R^2^), where R^2^ denotes the exposure variance determined using each IV and R^2^ = 2 × eaf × (1 − eaf) × beta^2^.^19^ IVs with F‐statistics less than 10 were considered weak instruments and were excluded from the MR analysis.^10^

#### MR analysis

Various statistical methods were employed to explore the causal associations between BC and the oral microbiome: inverse variance‐weighted (IVW) method,^20^ simple mode, weighted mode, weighted median (WM),^21^ and MR‐Egger regression.^22^ The IVW approach is a common and practical method when each IV conforms to the core tenets of MR, particularly the absence of horizontal pleiotropy and the provision of unbiased estimates. On the other hand, the WM method, which ascertains the central tendency of the effect estimates from all IVs, is a notable alternative, particularly when some IVs deviate from the MR prerequisites, including the presence of horizontal pleiotropy. In the MR‐Egger method, the causal effects are evaluated and horizontal pleiotropy is identified and adjusted; therefore, it is an indispensable tool when such pleiotropy is expected.^20^ Associations between variables were considered significant if the resulting *p*‐value of the IVW method was <0.05, with the estimated direction of the other four MR methods being consistent with that of IVW.^23^

#### Sensitivity analysis

Multiple analyses encompassing heterogeneity, pleiotropy, and leave‐one‐out sensitivity tests were employed. Cochrane's Q test was employed to evaluate comprehensive pleiotropy in the IVW MR findings, with a *p*‐value of 0.05 suggesting the presence of heterogeneity. The average horizontal pleiotropy of the IVs in MR‐Egger regression was determined using the intercept term and evaluating funnel plot asymmetry.^24^ Furthermore, MR‐PRESSO was utilized to detect the presence of pleiotropy and rectify horizontal pleiotropy by identifying and eliminating possible outliers. Thereafter, leave‐one‐out analysis was performed to determine if there were significant alterations in the causal effects before and after removing outliers.^23^ An established significance level of *p* < 0.05 suggested the presence of heterogeneity.

All projected effect sizes or odds ratios (ORs) were presented with corresponding 95% confidence intervals (CIs). All statistical tests were two‐tailed, and the associations were considered significant if the *p*‐value was <0.05. The open‐source statistical software R (version: 4.2.2) was used to conduct all analyses. The TwoSampleMR package (version: 0.5.6)^23^ was primarily used to conduct the analyses.

### Case–control study

The case–control study was conducted at Cancer Hospital (Chinese Academy of Medical Sciences) from January 2022 to March 2022. BC was diagnosed via a comprehensive evaluation, encompassing clinical examination, imaging studies, and histopathological confirmation, including fine needle aspiration or core needle biopsy of breast tissues. Patients who were less than 18 years of age, had a history of other malignant tumors, had other oral disorders, had received antibiotic or probiotic treatment within the last 2 months, and had incomplete data were excluded. Simultaneously, healthy controls, including those with benign breast diseases, were selected from the breast clinics of the same institution. The exclusion criteria for control participants were similar, with the addition of individuals with gastrointestinal disorders, a history of malignant tumors, chronic noncommunicable diseases, and incomplete data.

Essential information on demographic and clinical characteristics was collected from the participants, including age, body mass index, smoking and alcohol consumption habits, diabetes status, history of oral contraceptive use, and number of live births. In total, 124 participants (102 patients with BC and 22 healthy controls) satisfied the inclusion criteria. The study protocol was approved by the Ethics Committee of Cancer Hospital, Chinese Academy of Medical Sciences.

The Salivettes sampling device (Sarstedt) was used to collect salivary specimens immediately after the participants woke up (7–8 a.m.). These samples were stored at −80°C. The CTAB/SDS protocol was applied to meticulously isolate genomic DNA from the biological specimens. Its concentration and purity were rigorously assessed via 1% agarose gel electrophoresis. High‐fidelity PCR was performed to amplify multiple variable regions of the 16S rRNA gene, incorporating specific primers and barcodes for each region, including 16S V4: 515F‐806R. Then, the PCR products were uniformly mixed with loading buffer and subjected to 2% agarose gel electrophoresis for visualization. For sequencing, libraries were prepared using a PCR‐free sample preparation kit to prevent potential contamination. Fluorometric and bioanalyzer methods were used to verify the quality of these libraries.

The Illumina NovaSeq platform was used to perform high‐throughput sequencing, generating 250 bp paired‐end reads. The initial quality control steps included filtering based on read quality and assembly using overlapping read information.

The derived sequencing data were used to perform bioinformatics and statistical analyses. The DADA2 method in QIIME2 software was employed to denoise and acquire amplicon sequence variants (ASVs). Normalized ASV abundance tables formed the foundation for subsequent analyses. The Wilcoxon test was used to measure alpha diversity. Both Bray–Curtis (weighted) and compositional Jaccard (unweighted) distances were employed to measure beta diversity. Linear discriminant analysis effect size (LEfSe) was applied to identify the microbes associated with tumor status, with genera with a linear discriminant analysis (LDA) score of >2.5 identified as the differentiating genera. Thereafter, random forest (RF) analysis was conducted. Based on the RF results, receiver operating characteristic (ROC) analysis was performed using the pROC package. The R program (version 4.2.2) was used to perform all statistical analyses. A *p*‐value of <0.05 indicated statistical significance.

## RESULTS

### Selection of IVs

Table 1 summarizes the GWAS data information for exposure and outcome. After excluding palindromic SNPs, 8009 and 8426 SNPs associated with the salivary and tongue microbiomes, respectively, were identified at the suggested significance threshold of P < 5.0 × 10^−6^. Further refinement by excluding the SNPs affected by LD and palindromic structures resulted in the identification of 405 and 406 SNPs associated with the salivary and tongue microbiomes, respectively, for MR analysis. These SNPs spanned five taxonomic levels: phylum, class, order, family, and genus. Notably, the attributes of the oral microbiota at lower taxonomic levels may correlate with those at higher levels, indicating a potential SNP overlap. The F‐statistics of the IVs were 20.01–32.44, all significantly surpassing the threshold of 10; this suggests the absence of weak instrument bias. Moreover, validation using the PhenoScanner^25^ (http://www.phenoscanner.medschl.cam.ac.uk/) and PheWAS^26^ (PheWAS, https://gwas.mrcieu.ac.uk/phewas/) databases confirmed that none of the IVs were associated with diabetes, obesity, smoking status, or previous alcohol consumption. This observation affirms that the MR preconditions were duly satisfied.

**TABLE 1:** Summary of the GWAS included in this MR study.

Exposures/outcomes	Consortium	Ethnicity	Sample sizes	N. SNPs	Year
Oral microbiome	CNGBdb	East Asian	2948 Tongue *N* = 2017 Saliva *N*= 1914	Tongue *N* = 8426 Saliva *N* = 8009	2021
BC	BBJ	East Asian	89 731	8 872 152	2020

Abbreviations: BBJ, BioBank Japan Project; CNGBdb, China National GeneBank DataBase; GWAS, genome‐wide association studies; IVs, instrumental variables; MR, Mendelian randomization; SNPs, single nucleotide polymorphisms.

### Two‐sample MR analysis

Using the IVW method, we identified 30 tongue bacterial species and 37 salivary bacterial species that were significantly associated with BC. The other four methods employed to analyze the casual association of these oral microbiomes also achieved comparable results. Leave‐one‐out sensitivity analysis revealed that no individual SNP dominated the overall assessment. Cochran's Q statistics and horizontal pleiotropy test revealed no substantial heterogeneity among the selected SNPs.

Among the detected bacterial species, 14 and 18 bacterial genera in the tongue and saliva, respectively, were associated with BC, with six common genera. These overlapping genera were as follows: family *Pasteurellaceae* genus *Aggregatibacter*, family *Streptococcaceae* genus *Streptococcus*, family *Bacteroidaceae* genus *Prevotella*, family *Pasteurellacea* genus *Haemophilus*, family *Lachnospiraceae* genus *Oribacterium*, and family *Erysipelatoclostridiaceae* genus *Solobacterium*.

The number of SNPs connected with each of the six shared bacterial genera was between 6 and 23. Among these genera, the species *Oribacterium umgs 1411* from the tongue microbiome exhibited the most remarkable association with 18 SNPs, whereas *Aggregatibacter umgs 1480* in the salivary microbiome exhibited the most significant association with 23 SNPs.

**FIGURE 1:**
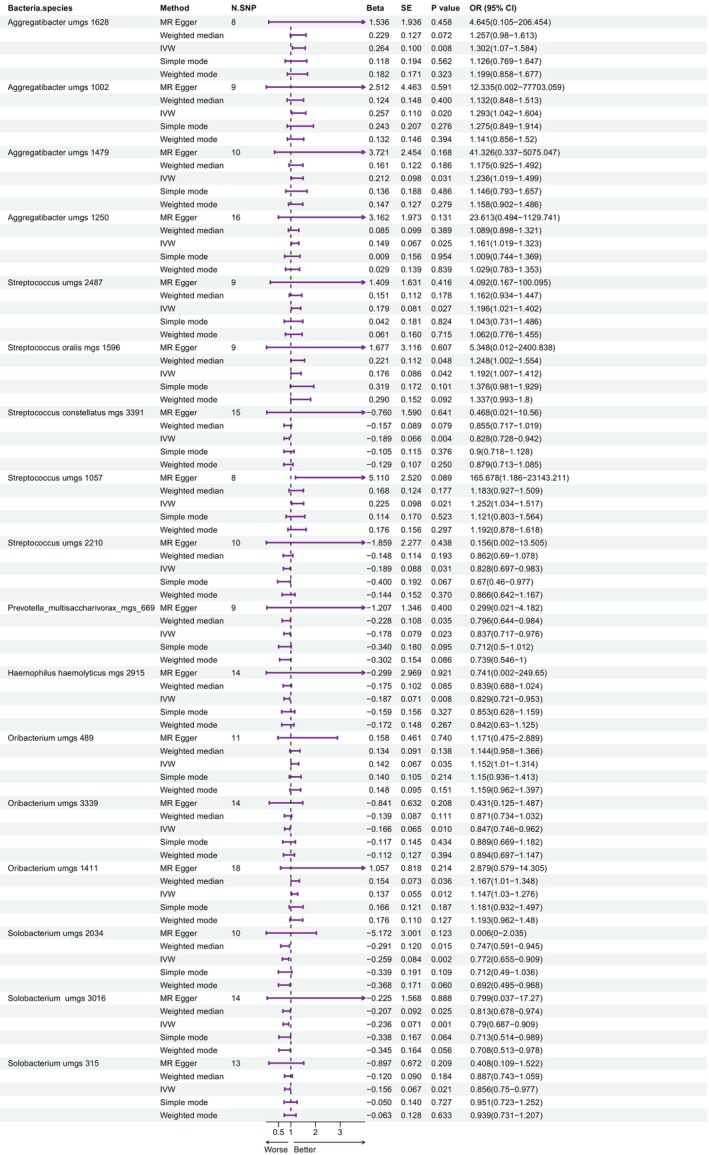
Forest plot showing the association between tongue microbiome species and breast cancer. IVW, inverse variance‐weighted; MR, Mendelian randomization; SNP, single nucleotide polymorphism.

In the tongue microbiome, the following genera were associated with an increased risk of BC: family *Pasteurellaceae* genus *Aggregatibacter* (with 1628, 1002, 1479, and 1250 having ORs of 1.302, 1.293, 1.236, and 1.161, respectively), family *Streptococcaceae* genus *Streptococcus umgs 2487* (OR = 1.196), *Streptococcus oralis mgs 1596* (OR = 1.192), *Streptococcus umgs 1057* (OR = 1.252), and family *Lachnospiraceae* genus *Oribacterium umgs 489* (OR = 1.152). Figure 1 illustrates a forest plot demonstrating the relationship between tongue microbiome species and BC risk.

**FIGURE 2:**
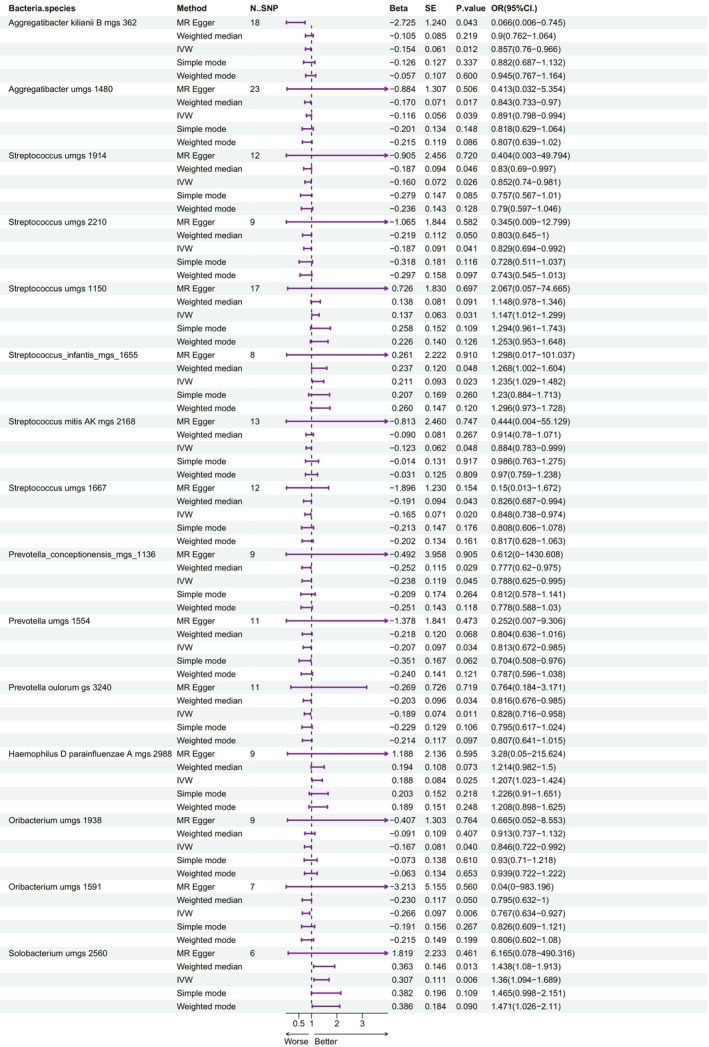
Forest plot showing the association between salivary microbiome species and breast cancer. IVW, inverse variance‐weighted; MR, Mendelian randomization; SNP, single nucleotide polymorphism.

For the salivary microbiome, the following were associated with an increased risk of BC: *Streptococcus umgs 1150* (OR = 1.147), *Streptococcus infantis mgs 1655* (OR = 1.235), *Haemophilus* (OR = 1.207), and *Solobacterium umgs 2560* (OR = 1.360). Figure 2 illustrates the corresponding forest plot demonstrating the association between salivary microbiome species and BC risk. Family *Bacteroidaceae* correlated with a decreased risk of BC in both the tongue and salivary microbiomes.

Supplementary Material, particularly Tables S1–S4, provides detailed data regarding the selected IVs. Furthermore, Figures S1–S12 illustrate the scatter diagrams demonstrating the computed causal effect sizes of the SNPs on the exposure (six microbiomes from both the tongue and saliva) and the result (BC). The STROBE‐MR checklist is in Table S5.

### Case–control study

In the case–control study, 124 participants (102 individuals diagnosed with BC and 22 healthy controls) were included. The baseline characteristics of both groups were similar. Table 2 comprehensively compares the baseline demographic and clinical characteristics of all participants.

**TABLE 2:** Clinical characteristics of patients in case control study.

Characteristics	BC (*N* = 102)	Controls (*N* = 22)	*p*‐value
Age, years Median (IQR)	49 (18.5)	54 (15.25)	0.163
BMI—kg/m^2^ Median (IQR)	24.46 (5.01)	24.8 (5.47)	0.460
Smoking status			0.793
Never smoker	97 (95.1%)	20 (90.91%)	
Former smoker	5 (4.9%)	2 (9.09%)	
Current smoker	0	0	
Alcohol consumption			0.933
Never drink	87 (85.29%)	18 (81.82%)	
<1 standard drink per day	15 (14.71%)	4 (18.18%)	
One standard drink per day	0	0	
Diabetes			0.464
Yes	61 (59.8%)	15 (68.18%)	
No	41 (40.2%)	7 (31.82%)	
Oral contraceptives use past			1.000
Yes	90 (88.24%)	19 (86.36%)	
No	12 (11.76%)	3 (13.64%)	
Number of live births			0.618
0	4 (3.92%)	1 (4.55%)	
1–2	57 (55.88%)	10 (45.45%)	
≥3	33 (32.35%)	8 (36.36%)	

Abbreviations: BMI, body mass index; IQR, interquartile range.

**FIGURE 3:**
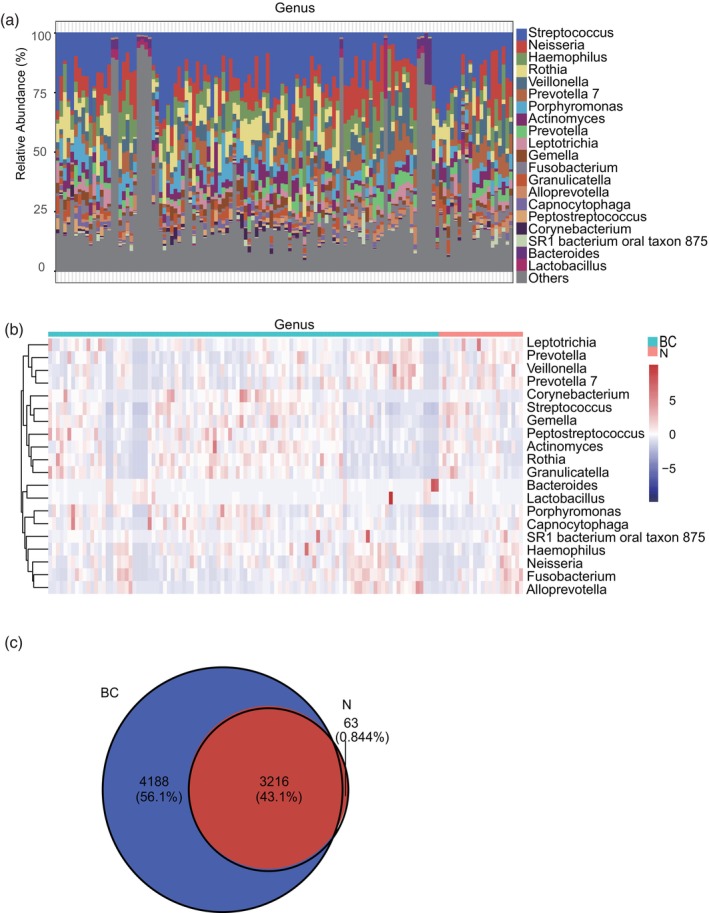
Differences in the composition and distribution of the oral microbiome in patients with breast cancer (BC) and healthy controls (N). (a) Bar chart showing the relative abundance of the bacterial taxa at the genus level. (b) Heat map showing species abundance clustering at the genus level. (c) Venn diagrams showing the composition of the different gut microbiome groups at the species level.

Differences in the oral microbiota were evaluated among the diverse groups. Figure 3a illustrates a bar plot revealing the proportion of community abundance at the genus level, highlighting microbiota structural analysis. The 20 most prevalent bacterial genera were identified as follows: *Streptococcus*, *Neisseria*, *Haemophilus*, *Rothia*, *Veillonella*, *Prevotella 7*, *Porphyromonas*, *Actinomyces*, *Prevotella*, *Leptotrichia*, *Gemella*, *Fusobacterium*, *Granulicatella*, *Alloprevotella*, *Capnocytophaga*, *Peptostreptococcus*, *Corynebacterium*, *SR1 bacterium oral taxon 875*, *Bacteroides*, and *Lactobacillus*. Figure 3b illustrates a heat map at the genus level, revealing a distinct difference in the relative abundance of specific bacterial genera between the patient (BC) and control (N) samples, concerning the top 20 bacterial genera. At the genus level, the abundance of genera such as *Streptococcus*, *Neisseria*, and *Haemophilus* was higher in BC tissues; in contrast, genera such as *Veillonella* and *Prevotella* were predominantly identified in control tissues, with a significantly decreased abundance in BC tissues. This highlights the differential bacterial composition in BC and normal control tissues. As shown in Figure 3c, the Venn diagram reveals the common and specific oral microbiota at the species level for each group. We observed that while a small set of genera was solely identified in the normal control group, most bacterial genera were shared between the BC and control groups, with a significant number also unique to the BC group.

**FIGURE 4:**
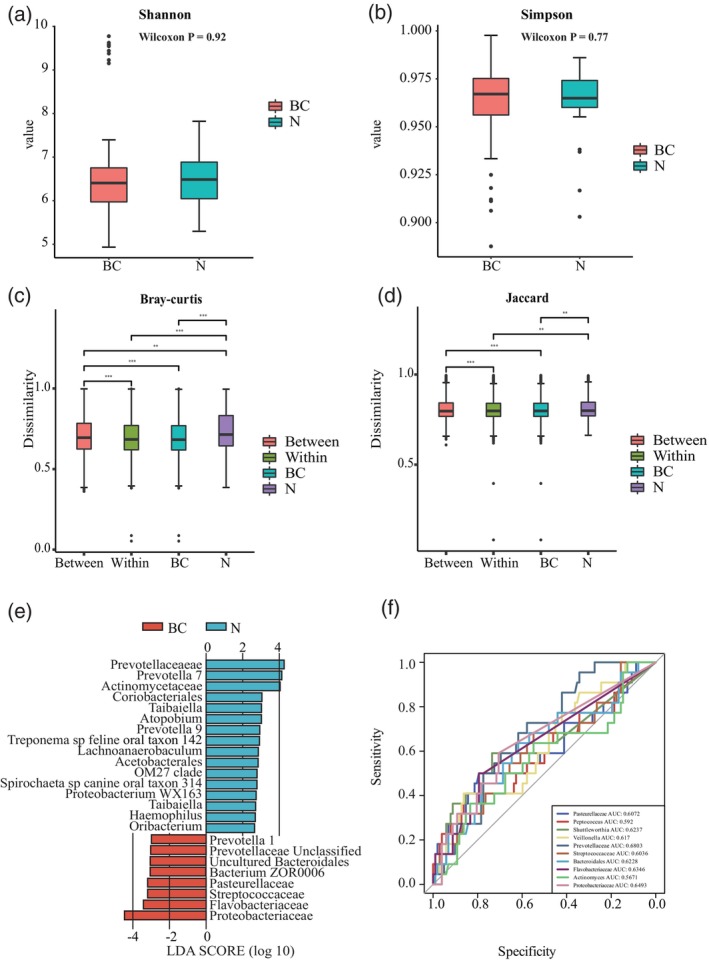
Relative abundances of the oral microbiome in patients with breast cancer (BC) and healthy controls (N). (a) Alpha diversities of the oral microbiota described using the Shannon index. (b) Alpha diversities described using the Simpson index. (c) Beta diversities of the oral microbiota based on Bray–Curtis distances. (d) Beta diversities based on Jaccard distance. (e) Linear discriminant analysis effect size analysis between the breast cancer (BC) and healthy control (N) groups. (f) Receiver operating characteristic (ROC) analysis of the top 10 genera in the BC and N groups.

Alpha and beta diversities serve as critical indicators for describing the overall constitution and distribution of the oral microbiota to BC risk. Alpha diversity is applied to compare the differences in species diversity among different groups. On the other hand, beta diversity compares the differences in species diversity between sample pairs using species distance metrics. Shannon and Simpson indexes were used to measure alpha diversity. Figure 4a,b illustrate no substantial difference in Shannon (*p* = 0.92) and Simpson (*p* = 0.77) indexes between patients with BC and healthy controls. However, for beta diversity, notable differences were observed in the Bray–Curtis (weighted) distance matrix and Jaccard (unweighted) distances at the genus level (Figure 4c, d, *p* < 0.05).

These results suggest significant differences in the abundance of several species between salivary samples from the BC and control groups. Subsequently, the oral microbiota may serve as a predictive biomarker for BC risk. LEfSe utilizes LDA to estimate the effect size of the abundance of each component (species) on the differences observed, thereby identifying the communities or species that significantly and differentially affect sample classification. An absolute LDA score of ≥2.5 was used as a criterion for significance. The larger the absolute LDA score associated with a differential bacterium, the higher the degree of difference between the groups. At the species level, LEfSe (Figure 4e) revealed significantly different strains among the groups; *Prevotellaceae*, *Pasteurellaceae*, *Streptococcaceae, Flavobacteriaceae*, *Proteobacteriaceae*, *Bacteroidales*, and *Bacterium ZOR0006* were identified as biomarkers for BC. Next, an RF model was trained using 10 biomarkers. The area under the curve (AUC) of the ROC curve was used to evaluate the performance of the constructed model. As per RF analysis, the AUC was 60.36%–68.03% for the mentioned bacterial species (Figure 4f). The *p*‐values for *Veillonella*, *Prevotellaceae*, *Streptococcaceae*, *Bacteroidales*, and *Proteobacteriaceae* are all less than 0.05, indicating strong predictive power for breast cancer with statistical significance. Among these, *Prevotellaceae* has the highest AUC value of 0.6803 (95%CI 0.573–0.788, *p* = 0.001, Table 3).

**TABLE 3:** Results of ROC curves for different bacterial species in predicting breast cancer.

Species	AUC	95% CI	SE	*p*‐value
Pasteurellaceae	0.6072	0.464–0.750	0.073	0.105
Peptococcus	0.5920	0.457–0.727	0.069	0.105
Shuttleworthia	0.6237	0.494–0.753	0.066	0.100
Veillonella	0.6170	0.525–0.709	0.047	0.032
Prevotellaceae	0.6803	0.573–0.788	0.055	0.001
Streptococcaceae	0.6036	0.488–0.719	0.059	0.005
Bacteroidales	0.6228	0.488–0.758	0.069	0.028
Flavobacteriaceae	0.6346	0.497–0.772	0.070	0.114
Actinomyces	0.5671	0.426–0.708	0.072	0.068
Proteobacteriaceae	0.6493	0.526–0.773	0.063	0.040

Abbreviations: AUC, area under the curve; CI, confidence interval; ROC, receiver operating characteristic; SE, standard error.

**FIGURE 5:**
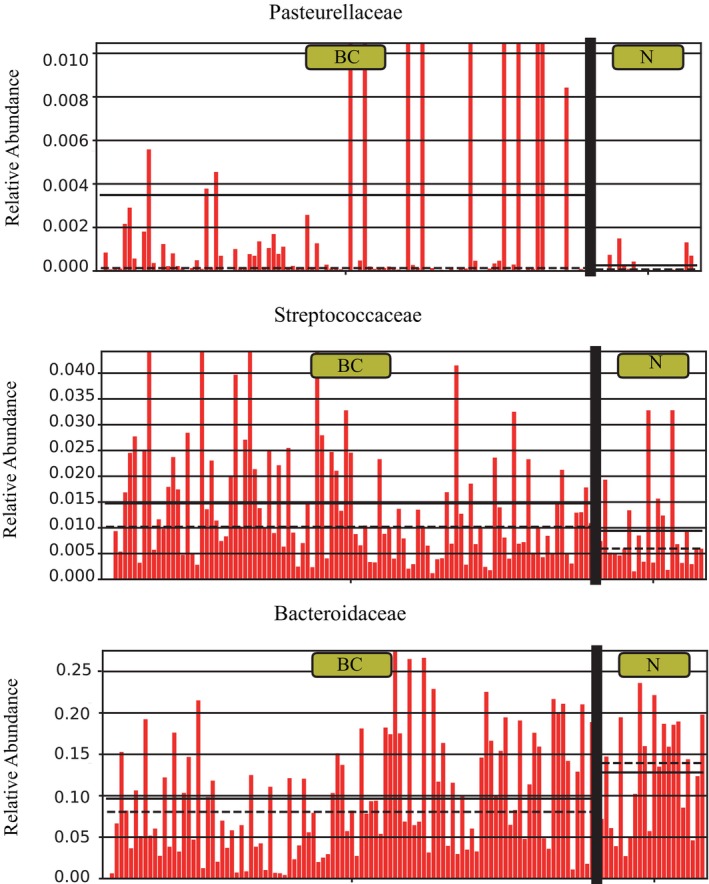
Bar plots to validate the Mendelian randomization (MR) results. Comparative relative abundance of the families *Pasteurellaceae* (a), *Streptococcaceae* (b), and *Bacteroidaceae* (c).

To corroborate the conclusions drawn via MR analysis, we determined the relative abundance of *Pasteurellaceae*, *Streptococcaceae*, and *Bacteroidaceae*. The relative abundance of *Pasteurellaceae* and *Streptococcaceae* was higher in the BC group than in the control group (Figure 5a, b). Simultaneously, the abundance of *Bacteroidaceae* was higher in the healthy control group (Figure 5c). These findings are consistent with the MR results. The abundance of the other families was not significantly different between both groups; this could be attributed to the limited sequencing data.

## DISCUSSION

Our study was a pioneering effort to apply MR to explore the causal association between the oral microbiome and BC. In this study, stringent quality control procedures were employed to prevent potential confounding factors and reverse causation during SNP selection. MR analysis revealed significant associations between 30 and 37 tongue and salivary bacteria species and BC incidence. By intersecting the tongue and salivary bacterial species findings, we identified six oral bacterial genera distributed across five families. Through our MR‐based research, we unveiled significant causal associations between the salivary and dorsal tongue microbiomes and BC. These relationships were further substantiated using sequencing data derived from our case–control study.

Recently, studies have continually revealed how microbial communities affect human health and diseases, particularly cancer. For example, a study has suggested that gut microbiome imbalance is associated with the occurrence of various cancers.^27^ Our findings extend this theory to the oral microbiome, particularly its association with BC risk. Specific bacterial genera, including *Pasteurellaceae* and *Streptococcaceae*, are relatively more abundant in patients with BC, possibly affecting cancer development via mechanisms that promote inflammation and immunoregulation. The significant causal relationship between the oral microbiome and BC adds a new dimension to our understanding of the multifactorial nature of cancer. The potential contribution of the oral microbiome to BC risk complements the findings from gut microbiome research, suggesting that microbial communities broadly affect systemic health and disease states, including cancer.

Studies on the effect of the oral microbiome on other cancer types, including colorectal and pancreatic cancers, have revealed the mechanisms that may also be associated with BC. For example, gut microbiome dysbiosis is associated with inflammation, DNA damage, and immune response alterations, which are key pathways in carcinogenesis. The effect of the oral microbiome on BC is mechanistically similar to the effect of the gut microbiome on other cancer types, including colorectal cancer. These include pathways that promote inflammatory responses, regulate immune surveillance, and affect hormone levels.^28^ Because the effect of the gut microbiome on colorectal cancer has been extensively researched, one identified mechanism is increasing cancer risk by promoting chronic inflammation,^29^ Similarly, specific bacteria in the oral microbiome, including *Porphyromonas gingivalis*, are associated with increased risks of inflammation and cancer.^30^ This commonality suggests that the inflammation‐promoting actions of different microbiomes can be a universal pathway affecting cancer development. Gut microbiota regulates the immune responses of the host, affecting cancer immune surveillance. Similarly, the oral microbiome, via similar mechanisms, may affect distant breast tissues by modulating cytokine activation and secretion by immune cells, affecting the risk of BC. While both oral and gut microbiomes can produce metabolites that affect host health, there can be differences in the specific metabolites produced. For example, short‐chain fatty acids (such as butyrate) produced by the gut microbiota confer a protective effect against colorectal cancer^31^; in contrast, some oral microbiota, including *Porphyromonas gingivalis*, produce proinflammatory metabolites such as lipopolysaccharides (LPS), which can travel to distant breast tissues via the bloodstream. In breast tissues, LPS binds to Toll‐like receptor 4 and activates the nuclear factor kappa B signaling pathway, promoting the release of inflammatory cytokines such as tumor necrosis factor‐α and interleukin (IL)‐6, thereby promoting cancer cell proliferation and survival.^32^

Species such as *Streptococcus* and *Haemophilus*, which predominantly colonize the oral cavity, may modulate immune responses.^33^, ^34^, ^35^ Notably, *Streptococcus salivarius* suppresses IL‐8 secretion.^34^ In contrast, *Haemophilus* is inversely associated with C‐reactive protein levels, a well‐known indicator of acute inflammation.^36^ *Haemophilus* and *Aggregatibacter*, members of the phylum Proteobacteria and family *Pasteurellaceae*, can trigger interferon‐γ secretion from leukocytes and T cells.^37^ Furthermore, the genus *Oribacterium* in the family *Lachnospi*ra*ceae* can help differentiate patients with liver and oral cavity cancers from healthy individuals; furthermore, this genus is associated with anti‐cyclic citrullinated peptide levels in patients with rheumatoid arthritis.^36^ The family *Bacteroidaceae*, belonging to the class Bacteroidia, plays a vital role in complex sugar fermentation,^38^ metabolic activities,^39^ and bile salt deconjugation.^40^ An increased abundance of *Bacteroidaceae* is associated with an increased risk of Alzheimer's disease, potentially owing to increased serum LPS levels.^41^ In the present study, we revealed that specific bacterial genera, including *Pasteurellacea*, *Streptococcaceae*, and *Lachnospi*ra*ceae*, are relatively more abundant in patients with BC. These observations are consistent with our findings, providing empirical support for how the oral microbiome affects BC risk by affecting the immune responses and inflammatory state of the host. In our case–control study, we further corroborated the conclusions of MR analysis, suggesting significant differences in the abundance of specific bacterial genera between the BC and healthy control groups. In particular, *Streptococcus*, *Neisseria*, and *Haemophilus* were more abundant in BC tissue samples, whereas *Veillonella* and *Prevotella* were primarily identified in control tissues but were significantly decreased in BC tissues. These bacterial composition differences highlight the variations between the oral microbial communities and BC tissues, potentially serving as predictive biomarkers for BC risk.

In a previous study, researchers identified a direct association between the microbial enrichment of *Fusobacterium*, a common species in the oral microbiota, and BC.^42^ Furthermore, a review in 2022 illustrated that oral microbes can colonize the lactiferous ducts of breast tissues by moving into systemic circulation.^43^ While previous studies have underscored a direct association between *Fusobacterium* enrichment and BC, our MR analysis did not confirm this association. Nevertheless, *Fusobacterium* was identified as one of the top 20 most prevalent bacterial genera in our study.

In addition, oral microbiota can affect hormone levels in the host via their metabolic activity, particularly estrogen metabolism. Enzymes such as β‐glucuronidase produced by some bacteria (*Bacteroides* and *Lactobacillus*) can affect estrogen recycling, increasing local or systemic estrogen levels, which, in turn, affects BC cell proliferation. Moreover, oral microbiota can produce bioactive small molecules, including peptides and polypeptides, that may travel to breast tissues via the bloodstream and directly or indirectly affect the behavior of cancer cells. For example, peptides produced by certain microbes may have functions similar to growth factors, promoting BC cell proliferation and migration. Furthermore, by affecting the local concentration of immune cells and inflammatory factors, the metabolic products of oral microbiota can promote the formation of a tumor microenvironment conducive to the growth and metastasis of cancer cells. This includes promoting angiogenesis, immune evasion, and extracellular matrix remodeling.

By comparing these mechanisms with those suggested for the effect of the oral microbiome on BC, researchers can identify the common and unique pathways via which microbiomes affect cancer risk. The commonality and differences in these mechanisms provide new directions for additional research, particularly in exploring how microbiomes affect cancer risk and progression via specific biomarkers and metabolic pathways. Gut microbiome modulation can decrease BC risk by altering estrogen metabolism and affecting immune responses.^44^ In particular, probiotics have demonstrated tumor inhibition potential and can be customized based on patient‐specific strain and dosage requirements.^45^, ^46^ Notably, microbiomes can affect the efficacy of chemo‐, immuno‐, and radiotherapies, with some bacteria such as *Lactobacillus* acidophilus capable of restoring the antitumor activity of platinum‐based drugs in germ‐free mice, thereby highlighting the potential of microbiome cocktails to enhance the effectiveness of conventional cancer treatments.^47^ Overall, microbes play a vital role in preventing and treating cancer. Analyzing the advantageous or unique members of the oral microbiome may propel new directions in bacteriotherapy.

This study possesses several robust aspects, including the application of the most recent GWAS data associated with the oral microbiome, application of MR to establish causal connections, and validation via 16S rRNA sequencing. Notably, MR analysis and validation were conducted on the East Asian population, adding to the depth of our findings.

However, our study had some limitations that should be acknowledged. First, the potential for horizontal pleiotropy may affect the selection of IVs in the MR process. Second, genetic predisposition, lifestyle habits, dietary variations, and environmental influences can affect the oral microbiome; this suggests that IVs only account for a small proportion of the variance; therefore, additional studies are warranted to investigate the complexity of alterations within the oral microbiome. Third, the marked disparity in sample sizes between the control and case groups can introduce potential bias. Therefore, caution should be exercised when extrapolating these findings to a broader population. Lastly, the study's focus on the East Asian population constrains the external validity of its conclusions.

Although we identified the potential causal associations between the oral microbiome and BC, we also highlighted the need for additional studies to bridge existing gaps. In the future, studies should aim to explore these associations in diverse populations and via longitudinal designs to understand how changes in the microbiome over time affect cancer risk and progression. Longitudinal studies will involve the enrollment of a diverse cohort of high‐risk and diagnosed individuals and the systematic sampling of their oral microbiomes during regular clinical evaluations. Their goals include characterizing baseline microbiome profiles, monitoring their changes at predetermined intervals, and correlating these changes with clinical outcomes. Furthermore, long‐term follow‐up should be emphasized to capture the comprehensive effect of the microbiome on BC, committing to data harmonization for enhanced validity and reproducibility among different populations. Moreover, mechanistic studies should delve into how specific microbes promote or inhibit tumor development by affecting the immune responses, metabolic pathways, and hormonal balance of the host. In addition, preclinical and clinical studies should investigate the potential effects of modulating the oral microbiome (e.g., probiotic supplementation) on decreasing BC risk. Such studies can facilitate the development of microbiome‐based diagnostics, preventive strategies, and adjuvant therapies that enhance the efficacy of conventional cancer therapies.

Collectively, integrating our study findings with existing research not only reinforces the significance of the microbiome in cancer research but also opens new interdisciplinary avenues for understanding and combating cancer. This holistic approach, considering both microbial and traditional cancer risk factors, promises to enrich the strategies for preventing, diagnosing, and treating cancer, leading to more personalized and effective healthcare solutions.

In conclusion, in this study, MR analysis presents supportive evidence for a potential causal association between the oral microbiome and BC, with partial validation accomplished via 16S rRNA sequencing. Oral microbes can play a significant role in preventing and treating BC, warranting additional investigation to entirely understand the underlying mechanisms.

## AUTHOR CONTRIBUTIONS

All authors contributed to the conception and design of the study. Kexin Feng contributed to the study design. Kexin Feng and Fei Ren contributed to the drafting of the article. Qingyao Shang contributed to data collection and imaging analysis. Xin Wang and Xiang Wang participated in data analysis and interpretation and led the revision of the article. All authors reviewed and approved the final manuscript.

## FUNDING INFORMATION

Not applicable.

## CONFLICT OF INTEREST STATEMENT

The authors declare that the research was conducted in the absence of any commercial or financial relationships that could be construed as a potential conflict of interest.

## Supporting information


**Data S1:** Supporting Information.

**Supplementary Table S3.** Detailed information about the IVs of the tongue dorsum microbiome.

**Supplementary Table S4.** Detailed information about the IVs of the saliva microbiome.

**Supplementary Table S5.** STROBE‐MR checklist of recommended items to address in reports of Mendelian randomization studies.^1,2^

## Data Availability

The datasets employed in the present study are accessible at the following website: https://db.cngb.org/search/project/CNP0001664. The original contributions presented in the 16S sequencing are publicly available at https://www.ncbi.nlm.nih.gov/sra/PRJNA1036382.
