# Supplementary material for: Association between oral microbiome and breast cancer in the east Asian population: A Mendelian randomization and case–control study
Source: Thorac Cancer. 2024 Mar 14;15(12):974–86. doi: 10.1111/1759-7714.15280 (PMC11045337; doi:10.1111/1759-7714.15280)
Supplement: Supplementary file 1 — Data S1: Supporting Information. [file TCA-15-974-s002.pdf]

Supplementary Material

1 Supplementary Figures and Tables

1.1 Supplementary Figures

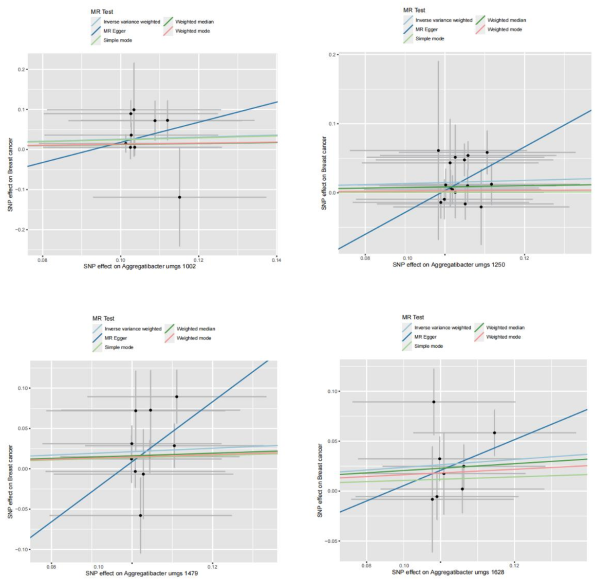

Supplementary Figure S1. Scatter plots of the association between *Genus_Aggregatibacter* of tongue microbiome and breast cancer.

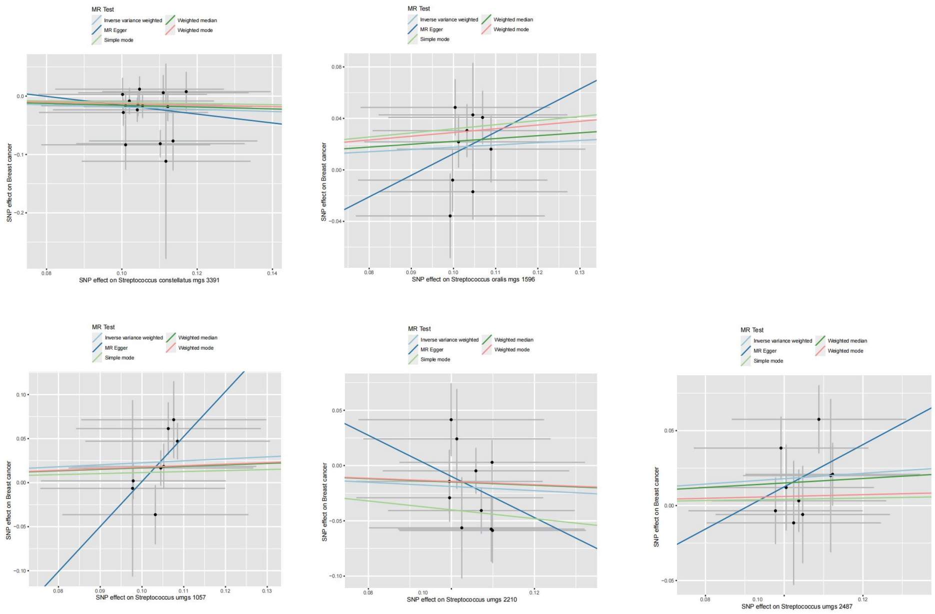

Supplementary Figure S2. Scatter plots of the association between *Genus_Streptococcus* of tongue microbiome and breast cancer.

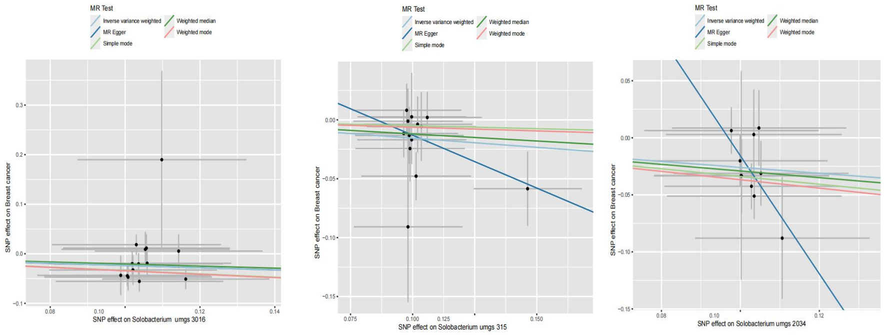

Supplementary Figure S3. Scatter plots of the association between *Genus_Solobacterium* of tongue microbiome and breast cancer.

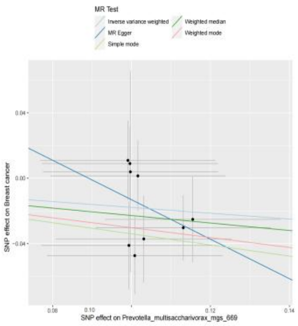

Supplementary Figure S4. Scatter plots of the Association Between *Genus_Prevotella* of tongue microbiome and breast cancer.

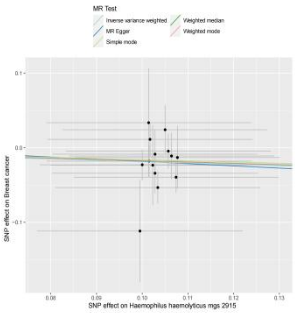

Supplementary Figure S5. Scatter plots of the association between *Genus_Haemophilus* of tongue microbiome and breast cancer.

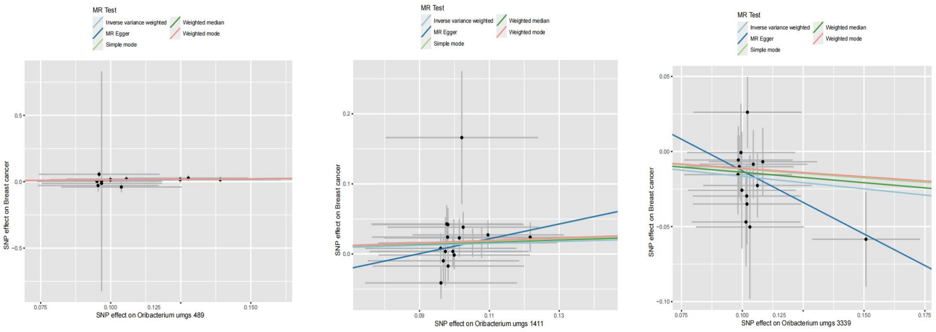

Supplementary Figure S6. Scatter plots of the association between *Genus_Oribacterium* of tongue microbiome and breast cancer.

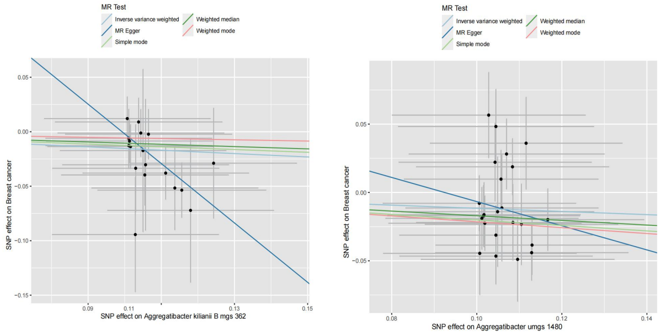

Supplementary Figure S7. Scatter plots of the association between *Genus_Aggregatibacte* of saliva microbiome and breast cancer.

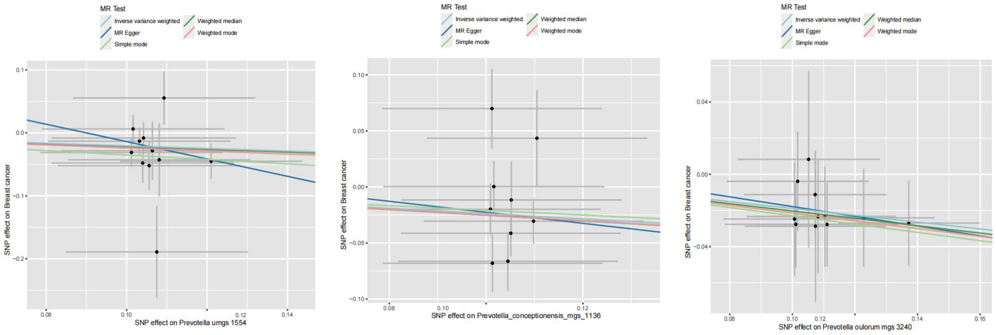

Supplementary Figure S8. Scatter plots of the association between *Genus_Prevotella* of saliva microbiome and breast cancer.

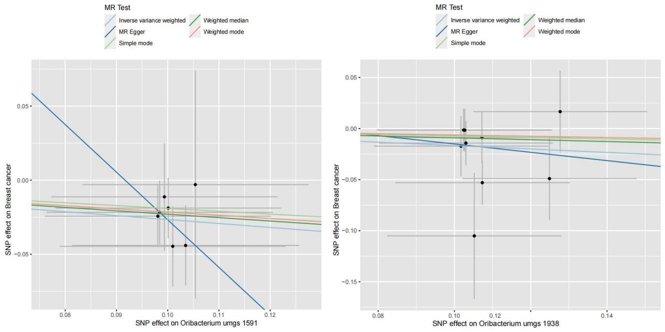

Supplementary Figure S9. Scatter plots of the association between *Genus_Oribacterium* of saliva microbiome and breast cancer.

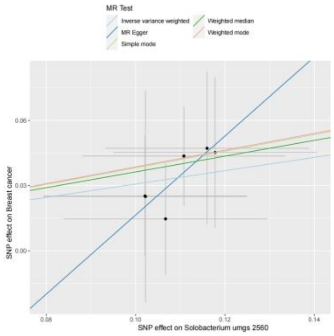

Supplementary Figure S10. Scatter plots of the association between *Genus_Solobacterium* of saliva microbiome and breast cancer.

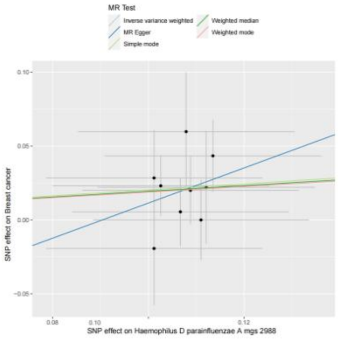

Supplementary Figure S11. Scatter plots of the association between *Genus_Haemophilus* of saliva microbiome and breast cancer.

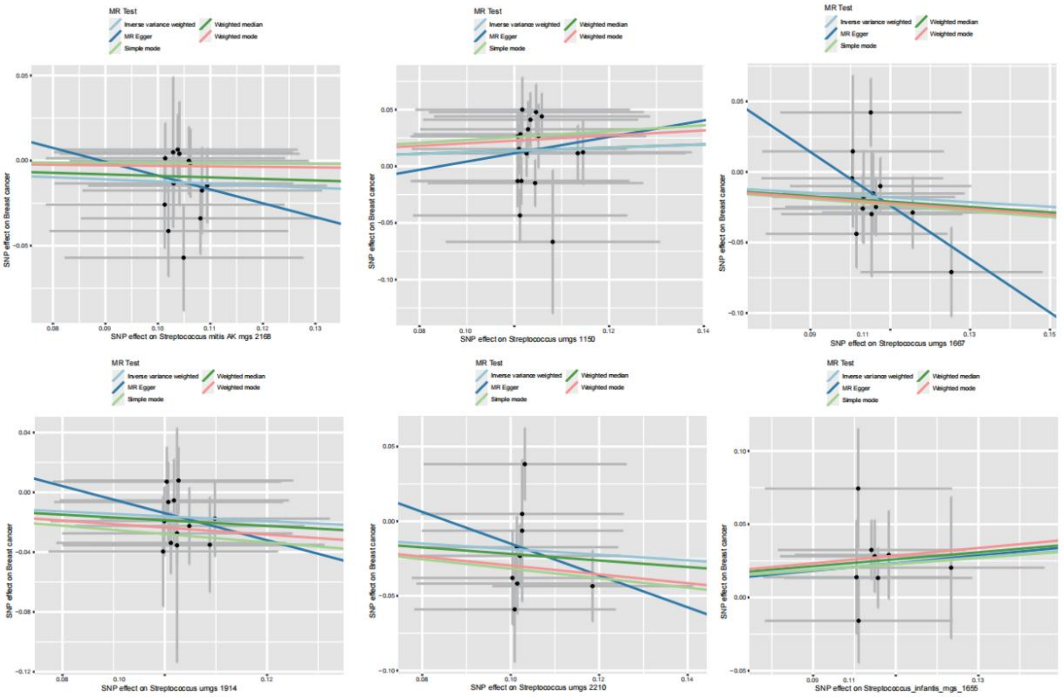

Supplementary Figure S12. Scatter plots of the association between *Genus_Streptococcus* of saliva microbiome and breast cancer.

1.2 Supplementary Tables

Table S1. The Association of Relative Abundance of Tongue Microbiome with Breast Cancer

	Bacteria species	Method	N. SNP	Beta	SE	P value	OR (95% CI)	R^2	F-statistics	Cochran's Q			Horizontal pleiotropy		
										Q	Q_df	Q_pval	Egger intercept	SE	P value
Aggregatibacter	Aggregatibacter umgs 1628	MR Egger	8	1.536	1.936	0.458	4.645(0.105-206.454)	0.087	23.620	8.101	6	0.231	-0.133	0.202	0.535
		Weighted median		0.229	0.127	0.072	1.257(0.98-1.613)								
		IVW		0.264	0.100	0.008	1.302(1.07-1.584)			8.686	7	0.276			
		Simple mode		0.118	0.194	0.562	1.126(0.769-1.647)								
		Weighted mode		0.182	0.171	0.323	1.199(0.858-1.677)								
	Aggregatibacter umgs 1002	MR Egger	9	2.512	4.463	0.591	12.335(0.002-77703.059)	0.112	24.946	8.701	7	0.275	-0.233	0.462	0.629
		Weighted median		0.124	0.148	0.400	1.132(0.848-1.513)								
		IVW		0.257	0.110	0.020	1.293(1.042-1.604)			9.019	8	0.341			
		Simple mode		0.243	0.207	0.276	1.275(0.849-1.914)								
		Weighted mode		0.132	0.146	0.394	1.141(0.856-1.52)								
	Aggregatibacter umgs 1479	MR Egger	10	3.721	2.454	0.168	41.326(0.337-5075.047)	0.109	24.106	8.839	8	0.356	-0.363	0.254	0.190

		Weighted median		0.161	0.122	0.186	1.175(0.925-1.492)								
		IVW		0.212	0.098	0.031	1.236(1.019-1.499)			11.101	9	0.269			
		Simple mode		0.136	0.188	0.486	1.146(0.793-1.657)								
		Weighted mode		0.147	0.127	0.279	1.158(0.902-1.486)								
		MR Egger		3.162	1.973	0.131	23.613(0.494-1129.741)			11.078	14	0.680	-0.313	0.205	0.149
		Weighted median	16	0.085	0.099	0.389	1.089(0.898-1.321)								
	Aggregatibacter umgs 1250	IVW		0.149	0.067	0.025	1.161(1.019-1.323)	0.187	26.732	13.411	15	0.571			
		Simple mode		0.009	0.156	0.954	1.009(0.744-1.369)								
		Weighted mode		0.029	0.139	0.839	1.029(0.783-1.353)								
		MR Egger		1.409	1.631	0.416	4.092(0.167-100.095)			5.852	7	0.557	-0.128	0.170	0.475
		Weighted median	9	0.151	0.112	0.178	1.162(0.934-1.447)								
	Streptococcus umgs 2487	IVW		0.179	0.081	0.027	1.196(1.021-1.402)	0.100	24.500	6.421	8	0.600			
Streptococcus		Simple mode		0.042	0.181	0.824	1.043(0.731-1.486)								
		Weighted mode		0.061	0.160	0.715	1.062(0.776-1.455)								
	Streptococcus oralis mgs 1596	MR Egger	9	1.677	3.116	0.607	5.348(0.012-2400.838)	0.096	23.215	8.007	6	0.238	0.454	0.276	0.151
		Weighted median		0.221	0.112	0.048	1.248(1.002-1.554)								

		IVW		0.176	0.086	0.042	1.192(1.007-1.412)			11.616	7	0.114			
		Simple mode		0.319	0.172	0.101	1.376(0.981-1.929)								
		Weighted mode		0.290	0.152	0.092	1.337(0.993-1.8)								
	Streptococcus constellatus mgs 3391	MR Egger	5	-0.760	1.590	0.641	0.468(0.021-10.56)	0.057	23.914	15.406	13	0.283	0.060	0.168	0.725
		Weighted median		-0.157	0.089	0.079	0.855(0.717-1.019)								
		IVW		-0.189	0.066	0.004	0.828(0.728-0.942)			15.559	14	0.341			
		Simple mode		-0.105	0.115	0.376	0.9(0.718-1.128)								
		Weighted mode		-0.129	0.107	0.250	0.879(0.713-1.085)								
	Streptococcus umgs 1057	MR Egger	8	5.110	2.520	0.089	165.678(1.186-23143.211)	0.098	24.036	4.633	6	0.592	-0.510	0.263	0.100
		Weighted median		0.168	0.124	0.177	1.183(0.927-1.509)								
		IVW		0.225	0.098	0.021	1.252(1.034-1.517)			8.395	7	0.299			
		Simple mode		0.114	0.170	0.523	1.121(0.803-1.564)								
		Weighted mode		0.176	0.156	0.297	1.192(0.878-1.618)								
	Streptococcus umgs 2210	MR Egger	10	-1.859	2.277	0.438	0.156(0.002-13.505)	0.123	25.261	10.653	8	0.222	0.176	0.240	0.484
		Weighted median		-0.148	0.114	0.193	0.862(0.69-1.078)								
		IVW		-0.189	0.088	0.031	0.828(0.697-0.983)			11.370	9	0.251			
		Simple mode		-0.400	0.192	0.067	0.67(0.46-0.977)								

		Weighted mode		-0.144	0.152	0.370	0.866(0.642-1.167)								
Prevotella	Prevotella_multisaccharivorax_mgs_669	MR Egger	9	-1.207	1.346	0.400	0.299(0.021-4.182)	0.098	23.829	6.316	7	0.503	0.108	0.140	0.469
		Weighted median		-0.228	0.108	0.035	0.796(0.644-0.984)								
		IVW		-0.178	0.079	0.023	0.837(0.717-0.976)			6.902	8	0.547			
		Simple mode		-0.340	0.180	0.095	0.712(0.5-1.012)								
		Weighted mode		-0.302	0.154	0.086	0.739(0.546-1)								
Haemophilus	Haemophilus haemolyticus mgs 2915	MR Egger	14	-0.299	2.969	0.921	0.741(0.002-249.65)	0.150	24.626	10.429	12	0.578	0.012	0.307	0.971
		Weighted median		-0.175	0.102	0.085	0.839(0.688-1.024)								
		IVW		-0.187	0.071	0.008	0.829(0.721-0.953)			10.431	13	0.658			
		Simple mode		-0.159	0.156	0.327	0.853(0.628-1.159)								
		Weighted mode		-0.172	0.148	0.267	0.842(0.63-1.125)								
Oribacterium	Oribacterium umgs 489	MR Egger	11	0.158	0.461	0.740	1.171(0.475-2.889)	0.150	28.978	7.882	9	0.546	-0.002	0.052	0.972
		Weighted median		0.134	0.091	0.138	1.144(0.958-1.366)								
		IVW		0.142	0.067	0.035	1.152(1.01-1.314)			7.883	10	0.640			
		Simple mode		0.140	0.105	0.214	1.15(0.936-1.413)								
		Weighted mode		0.148	0.095	0.151	1.159(0.962-1.397)								

	Oribacterium umgs 3339	MR Egger	14	-0.841	0.632	0.208	0.431(0.125-1.487)	0.159	26.826	6.786	12	0.871	0.071	0.066	0.304
		Weighted median		-0.139	0.087	0.111	0.871(0.734-1.032)								
		IVW		-0.166	0.065	0.010	0.847(0.746-0.962)			7.940	13	0.847			
		Simple mode		-0.117	0.145	0.434	0.889(0.669-1.182)								
		Weighted mode		-0.112	0.127	0.394	0.894(0.697-1.147)								
	Oribacterium umgs 1411	MR Egger	18	1.057	0.818	0.214	2.879(0.579-14.305)	0.198	27.056	15.707	16	0.474	-0.095	0.084	0.276
		Weighted median		0.154	0.073	0.036	1.167(1.01-1.348)								
		IVW		0.137	0.055	0.012	1.147(1.03-1.276)			16.979	17	0.456			
		Simple mode		0.166	0.121	0.187	1.181(0.932-1.497)								
		Weighted mode		0.176	0.110	0.127	1.193(0.962-1.48)								
Solobacterium	Solobacterium umgs 2034	MR Egger	10	-5.172	3.001	0.123	0.006(0-2.035)	0.108	23.975	5.009	8	0.757	0.501	0.306	0.140
		Weighted median		-0.291	0.120	0.015	0.747(0.591-0.945)								
		IVW		-0.259	0.084	0.002	0.772(0.655-0.909)			7.691	9	0.566			
		Simple mode		-0.339	0.191	0.109	0.712(0.49-1.036)								
		Weighted mode		-0.368	0.171	0.060	0.692(0.495-0.968)								
	Solobacterium umgs 3016	MR Egger	14	-0.225	1.568	0.888	0.799(0.037-17.27)	0.154	25.711	15.797	12	0.201	-0.001	0.165	0.994
		Weighted median		-0.207	0.092	0.025	0.813(0.678-0.974)								

		IVW		-0.236	0.071	0.001	0.79(0.687-0.909)			15.797	13	0.260			
		Simple mode		-0.338	0.167	0.064	0.713(0.514-0.989)								
		Weighted mode		-0.345	0.164	0.056	0.708(0.513-0.978)								
	Solobacterium umgs 315	MR Egger	13	-0.897	0.672	0.209	0.408(0.109-1.522)	0.146	26.222	6.866	11	0.810	0.077	0.069	0.291
		Weighted median		-0.120	0.090	0.184	0.887(0.743-1.059)								
		IVW		-0.156	0.067	0.021	0.856(0.75-0.977)			8.097	12	0.777			
		Simple mode		-0.050	0.140	0.727	0.951(0.723-1.252)								
		Weighted mode		-0.063	0.128	0.633	0.939(0.731-1.207)								

SNP, Single Nucleotide Polymorphism; SE, Standard Error; OR, Odds Ratio; CI, Confidence Interval; MR, Mendelian Randomization; IVW, Inverse Variance Weighted; Q_df, degrees of freedom of Cochran's Q

Table S2. The Association of Relative Abundance of Saliva Microbiome with Breast Cancer

	Bacteria species	Method	N. SNP	Beta	SE	P value	OR (95% CI)	R^2	F-statistics	Cochran's Q		Horizontal pleiotropy			
										Q	Q_df	Q_pval	Egger intercept	SE	P value
Aggregatibacter	Aggregatibacter kilianii B mgs 362	MR Egger	18	-2.725	1.240	0.043	0.066(0.006-0.745)	0.208	27.330	7.379	16	0.965	0.271	0.130	0.054
		Weighted median		-0.105	0.085	0.219	0.9(0.762-1.064)								
		IVW		-0.154	0.061	0.012	0.857(0.76-0.966)			11.685	17	0.819			
		Simple mode		-0.126	0.127	0.337	0.882(0.687-1.132)								

		Weighted mode		-0.057	0.107	0.600	0.945(0.767-1.164)								
	Aggregatibacter umgs 1480	MR Egger	23	-0.884	1.307	0.506	0.413(0.032-5.354)	0.262	28.961	29.187	21	0.110	0.082	0.139	0.563
		Weighted median		-0.170	0.071	0.017	0.843(0.733-0.97)								
		IVW		-0.116	0.056	0.039	0.891(0.798-0.994)			29.667	22	0.127			
		Simple mode		-0.201	0.134	0.148	0.818(0.629-1.064)								
		Weighted mode		-0.215	0.119	0.086	0.807(0.639-1.02)								
Streptococcus	Streptococcus umgs 1914	MR Egger	12	-0.905	2.456	0.720	0.404(0.003-49.794)	0.133	24.065	4.295	10	0.933	0.077	0.252	0.768
		Weighted median		-0.187	0.094	0.046	0.83(0.69-0.997)								
		IVW		-0.160	0.072	0.026	0.852(0.74-0.981)			4.387	11	0.957			
		Simple mode		-0.279	0.147	0.085	0.757(0.567-1.01)								
		Weighted mode		-0.236	0.143	0.128	0.79(0.597-1.046)								
	Streptococcus umgs 2210	MR Egger	9	-1.065	1.844	0.582	0.345(0.009-12.799)	0.098	22.771	10.444	7	0.165	0.091	0.192	0.648
		Weighted median		-0.219	0.112	0.050	0.803(0.645-1)								
		IVW		-0.187	0.091	0.041	0.829(0.694-0.992)			10.783	8	0.214			
		Simple mode		-0.318	0.181	0.116	0.728(0.511-1.037)								

		Weighted mode		-0.297	0.158	0.097	0.743(0.545-1.013)								
	Streptococcus umgs 1150	MR Egger	17	0.726	1.830	0.697	2.067(0.057-74.665)	0.200	26.202	20.917	15	0.139	-0.061	0.190	0.752
		Weighted median		0.138	0.081	0.091	1.148(0.978-1.346)								
		IVW		0.137	0.063	0.031	1.147(1.012-1.299)			21.062	16	0.176			
		Simple mode		0.258	0.152	0.109	1.294(0.961-1.743)								
		Weighted mode		0.226	0.140	0.126	1.253(0.953-1.648)								
	Streptococcus _infantis_mgs _1655	MR Egger	8	0.261	2.222	0.910	1.298(0.017-101.037)	0.092	24.043	3.984	6	0.679	-0.005	0.234	0.983
		Weighted median		0.237	0.120	0.048	1.268(1.002-1.604)								
		IVW		0.211	0.093	0.023	1.235(1.029-1.482)			3.984	7	0.782			
		Simple mode		0.207	0.169	0.260	1.23(0.884-1.713)								
		Weighted mode		0.260	0.147	0.120	1.296(0.973-1.728)								
	Streptococcus mitis AK mgs 2168	MR Egger	13	-0.813	2.460	0.747	0.444(0.004-55.129)	0.143	24.409	6.843	11	0.812	0.072	0.258	0.784
		Weighted median		-0.090	0.081	0.267	0.914(0.78-1.071)								
		IVW		-0.123	0.062	0.048	0.884(0.783-0.999)			6.921	12	0.863			
		Simple mode		-0.014	0.131	0.917	0.986(0.763-1.275)								
		Weighted mode		-0.031	0.125	0.809	0.97(0.759-1.238)								
		MR Egger	12	-1.896	1.230	0.154	0.15(0.013-1.672)	0.139	25.358	9.277	10	0.506	0.185	0.131	0.189

	Streptococcus umgs 1667	Weighted median		-0.191	0.094	0.043	0.826(0.687-0.994)								
		IVW		-0.165	0.071	0.020	0.848(0.738-0.974)			11.266	11	0.421			
		Simple mode		-0.213	0.147	0.176	0.808(0.606-1.078)								
		Weighted mode		-0.202	0.134	0.161	0.817(0.628-1.063)								
Prevotella	Prevotella_conceptionensis_mgs_1136	MR Egger	9	-0.492	3.958	0.905	0.612(0-1430.608)	0.099	23.189	17.384	7	0.015	0.027	0.413	0.951
		Weighted median		-0.252	0.115	0.029	0.777(0.62-0.975)								
		IVW		-0.238	0.119	0.045	0.788(0.625-0.995)			17.394	8	0.026			
		Simple mode		-0.209	0.174	0.264	0.812(0.578-1.141)								
		Weighted mode		-0.251	0.143	0.118	0.778(0.588-1.03)								
	Prevotella umgs 1554	MR Egger	11	-1.378	1.841	0.473	0.252(0.007-9.306)	0.138	25.204	12.347	9	0.194	0.124	0.194	0.540
		Weighted median		-0.218	0.120	0.068	0.804(0.636-1.016)								
		IVW		-0.207	0.097	0.034	0.813(0.672-0.985)			12.904	10	0.229			
		Simple mode		-0.351	0.167	0.062	0.704(0.508-0.976)								
		Weighted mode		-0.240	0.141	0.121	0.787(0.596-1.038)								
	Prevotella oulorum gs 3240	MR Egger	11	-0.269	0.726	0.719	0.764(0.184-3.171)	0.137	27.218						
		Weighted median		-0.203	0.096	0.034	0.816(0.676-0.985)								
		IVW		-0.189	0.074	0.011	0.828(0.716-0.958)								

		Simple mode		-0.229	0.129	0.106	0.795(0.617-1.024)								
		Weighted mode		-0.214	0.117	0.097	0.807(0.641-1.015)								
		MR Egger		1.188	2.136	0.595	3.28(0.05-215.624)			3.686	7	0.815	-0.107	0.229	0.654
		Weighted median		0.194	0.108	0.073	1.214(0.982-1.5)								
Haemophilus	Haemophilus D parainfluenzae A mgs 2988	IVW	9	0.188	0.084	0.025	1.207(1.023-1.424)	0.118	25.123	3.906	8	0.866			
		Simple mode		0.203	0.152	0.218	1.226(0.91-1.651)								
		Weighted mode		0.189	0.151	0.248	1.208(0.898-1.625)								
		MR Egger		-0.407	1.303	0.764	0.665(0.052-8.553)			7.371	7	0.391	0.026	0.139	0.858
		Weighted median		-0.091	0.109	0.407	0.913(0.737-1.132)								
	Oribacterium umgs 1938	IVW	9	-0.167	0.081	0.040	0.846(0.722-0.992)	0.108	25.465	7.407	8	0.493			
		Simple mode		-0.073	0.138	0.610	0.93(0.71-1.218)								
		Weighted mode		-0.063	0.134	0.653	0.939(0.722-1.222)								
Oribacterium		MR Egger		-3.213	5.155	0.560	0.04(0-983.196)			0.974	5	0.965	0.295	0.515	0.592
		Weighted median		-0.230	0.117	0.050	0.795(0.632-1)								
	Oribacterium umgs 1591	IVW	7	-0.266	0.097	0.006	0.767(0.634-0.927)	0.088	22.793	1.301	6	0.972			
		Simple mode		-0.191	0.156	0.267	0.826(0.609-1.121)								
		Weighted mode		-0.215	0.149	0.199	0.806(0.602-1.08)								

Solobacterium	Solobacterium umgs 2560	MR Egger	6	1.819	2.233	0.461	6.165(0.078-490.316)	0.072	24.628	0.456	4	0.978	-0.165	0.244	0.535
		Weighted median		0.363	0.146	0.013	1.438(1.08-1.913)								
		IVW		0.307	0.111	0.006	1.36(1.094-1.689)			0.916	5	0.969			
		Simple mode		0.382	0.196	0.109	1.465(0.998-2.151)								
		Weighted mode		0.386	0.184	0.090	1.471 (1.026-2.11)								

SNP, Single Nucleotide Polymorphism; SE, Standard Error; OR, Odds Ratio; CI, Confidence Interval; MR, Mendelian Randomization; IVW, Inverse Variance Weighted; Q_df, degrees of freedom of Cochran's Q
