# Supplementary material for: Association between oral microbiome and breast cancer in the east Asian population: A Mendelian randomization and case–control study
Source: Thorac Cancer. 2024 Mar 14;15(12):974–86. doi: 10.1111/1759-7714.15280 (PMC11045337; doi:10.1111/1759-7714.15280)
Supplement: Supplementary file 4 — Supplementary Table S5. STROBE‐MR checklist of recommended items to address in reports of Mendelian randomization studies.1,2 [file TCA-15-974-s003.docx]

Supplementary Table S5. STROBE-MR checklist of recommended items to address in reports of Mendelian randomization studies^1^ ^2^

| **Item No.** | **Section** | **Checklist item** | **Page No.** | **Relevant text from manuscript** |
| --- | --- | --- | --- | --- |
| 1 | **TITLE and ABSTRACT** | Indicate Mendelian randomization (MR) as the study’s design in the title and/or the abstract if that is a main purpose of the study | 1 | Title: Association between the Oral Microbiome and Breast Cancer in an Asian Population: A Mendelian Randomization Study.  The abstract also describe the study as MR |
|  | **INTRODUCTION** |  |  |  |
| 2 | **Background** | Explain the scientific background and rationale for the reported study. What is the exposure? Is a potential causal relationship between exposure and outcome plausible? Justify why MR is a helpful method to address the study question | 2 | The exposure in this article includes the oral microbiome, and the outcome of interest is breast cancer. Some studies have confirmed that these factors can influence breast cancer, but there is currently a lack of evidence for an association between the aforementioned exposures and breast cancer. Therefore, MR (Mendelian Randomization) is used to investigate whether there is an association between the above exposures and breast cancer, ultimately aiming to prevent or delay the progression of the disease. |
| 3 | **Objectives** | State specific objectives clearly, including pre-specified causal hypotheses (if any). State that MR is a method that, under specific assumptions, intends to estimate causal effects | 2 | The causative relationship between BC and the oral microbiome remains unclear. The objective of this study was to thoroughly examine the relationship between oral microbiota and BC in the East Asian population, using a two-sample Mendelian randomization (MR) analysis alongside a case-control study. |
|  | **METHODS** |  |  |  |
| 4 | **Study design and data sources** | Present key elements of the study design early in the article. Consider including a table listing sources of data for all phases of the study. For each data source contributing to the analysis, describe the following: |  |  |
|  | a) | Setting: Describe the study design and the underlying population, if possible. Describe the setting, locations, and relevant dates, including periods of recruitment, exposure, follow-up, and data collection, when available. | 2 and 12 | The article describes the use of data from genome-wide association studies (GWAS) on oral microbiomes and breast cancer in East Asian populations in its data sources. Table 1 shows the information on the sources of exposure and outcome data. |
|  | b) | Participants: Give the eligibility criteria, and the sources and methods of selection of participants. Report the sample size, and whether any power or sample size calculations were carried out prior to the main analysis | 2 and 12 | Following stringent quality control measures, a total of 2984 individuals (2017 tongue dorsum and 1915 salivary) were included. Regarding BC, the data employed in this study were extracted from a comprehensive GWAS performed on an Asian population. This dataset comprised 5552 BC instances and 89731 control cases. Table 1 shows the information on the sources of exposure and outcome data.  The method for calculating the sample size was not reported. |
|  | c) | Describe measurement, quality control and selection of genetic variants | 3 | Page 3 reports the screening process and criteria for genetic variant instrumental variables, with more detailed information provided in Supplementary Tables 3 and 4. |
|  | d) | For each exposure, outcome, and other relevant variables, describe methods of assessment and diagnostic criteria for diseases | 3 | The Method describes the definition of outcomes in its methodology section and provides references that the definition is based on. |
|  | e) | Provide details of ethics committee approval and participant informed consent, if relevant | 10 | The data comes from public databases, and the sources of the data have been clearly stated. The data sets employed in the present investigation are accessible at the following website: https://db.cngb.org/search/project/CNP0001664. |
| 5 | **Assumptions** | Explicitly state the three core IV assumptions for the main analysis (relevance, independence and exclusion restriction) as well assumptions for any additional or sensitivity analysis | 3 | The article does not provide detailed descriptions of the three core assumptions but reflects these core assumptions in the analysis methods. Additionally, it describes three sensitivity analysis methods and methods for detecting horizontal pleiotropy: the weighted median method, MR-Egger, and MR-PRESSO. It also reports the use of F-statistics to calculate statistical power and Q-statistics to detect heterogeneity. |
| 6 | **Statistical methods: main analysis** | Describe statistical methods and statistics used |  |  |
|  | a) | Describe how quantitative variables were handled in the analyses (i.e., scale, units, model) | 3 | When the number of genetic variant instrumental variables exceeds three, a random effects model is used for analysis; otherwise, a fixed effects model is adopted. The statistical effect sizes or measurement units for exposure, outcomes, and related covariates have not been converted, and therefore are not reported. |
|  | b) | Describe how genetic variants were handled in the analyses and, if applicable, how their weights were selected | 2 | Following stringent quality control measures, a total of 2984 individuals (2017 tongue dorsum and 1915 salivary) were included, and nearly 10 million common and low-frequency variants (MAF ≥ 0.5%) were retained. Additional, comprehensive information regarding sample acquisition, sequencing protocols, microbiome trait preparation, and observational and genotyping analyses can be referred to in the article. |
|  | c) | Describe the MR estimator (e.g. two-stage least squares, Wald ratio) and related statistics. Detail the included covariates and, in case of two-sample MR, whether the same covariate set was used for adjustment in the two samples | 3 | The article reports that the genetic instrumental variables related to exposure come from the analysis results of a GWAS. The study population consisted of 3,932 oral samples (2,017 from the tongue dorsum and 1,915 from saliva) collected from 2,984 adult Chinese individuals. The genetic model did not adjust for factors such as age, gender, and study location. Additionally, the text provides a detailed description of the specific MR statistical methods and the software used for analysis. |
|  | d) | Explain how missing data were addressed | 3 | The article reports the methods for handling missing values. |
|  | e) | If applicable, indicate how multiple testing was addressed | 3 | This article does not involve multiple testing. |
| 7 | **Assessment of assumptions** | Describe any methods or prior knowledge used to assess the assumptions or justify their validity | 3 | This article reports the use of F-statistics to estimate statistical power and does not use other methods. |
| 8 | **Sensitivity analyses and additional analyses** | Describe any sensitivity analyses or additional analyses performed (e.g. comparison of effect estimates from different approaches, independent replication, bias analytic techniques, validation of instruments, simulations) | 3 | This article employs three methods for sensitivity and horizontal pleiotropy detection: MR-Egger, MR-PRESSO, and leave-one-out assessment. It uses F-statistics to evaluate statistical power, Q-statistics to detect heterogeneity, and calculates the False Discovery Rate (FDR). |
| 9 | **Software and pre-registration** |  |  |  |
|  | a) | Name statistical software and package(s), including version and settings used | 3 | All examinations were undertaken using the open-source statistical software R (version: 4.2.2). The R packages utilized include TwoSampleMR, MR-PRESSO, and Mendelian Randomization, all of which are available for free on the official R software website. |
|  | b) | State whether the study protocol and details were pre-registered (as well as when and where) |  | This study was not registered. |
|  | **RESULTS** |  |  |  |
| 10 | **Descriptive data** |  |  |  |
|  | a) | Report the numbers of individuals at each stage of included studies and reasons for exclusion. Consider use of a flow diagram |  | The article provides the number of the sample population in the methodology section, but does not provide information about the population in the results. Information on the excluded population and reasons for exclusion were not provided, nor was a flowchart used. |
|  | b) | Report summary statistics for phenotypic exposure(s), outcome(s), and other relevant variables (e.g. means, SDs, proportions) |  | Supplementary Tables 1-4 provide relevant information, detailing the characteristics of the study subjects, exposure, and potential confounding factors. |
|  | c) | If the data sources include meta-analyses of previous studies, provide the assessments of heterogeneity across these studies |  | The data sources for this study do not include meta-analysis. |
|  | d) | For two-sample MR:  i.  Provide justification of the similarity of the genetic variant-exposure associations between the exposure and outcome samples  ii.  Provide information on the number of individuals who overlap between the exposure and outcome studies |  | This article is a two-sample MR study. Table 1 lists all sample populations as originating from Asia, hence there is minimal racial heterogeneity. The article does not report information on overlapping populations, nor are there overlapping subjects, therefore such details are not reported. |
| 11 | **Main results** |  |  |  |
|  | a) | Report the associations between genetic variant and exposure, and between genetic variant and outcome, preferably on an interpretable scale |  | Supplementary Tables 1 and 2 reported the content of the project, including the number of instrumental variable SNPs, sample size, adjusted confounding factors, the association between exposure and instrumental variables, and statistical power. |
|  | b) | Report MR estimates of the relationship between exposure and outcome, and the measures of uncertainty from the MR analysis, on an interpretable scale, such as odds ratio or relative risk per SD difference |  | Supplementary Tables 1 and 2 reported the content of the project, including MR estimates, OR value and 95%CI. |
|  | c) | If relevant, consider translating estimates of relative risk into absolute risk for a meaningful time period |  | Due to the lack of baseline incidence data for breast cancer in specific populations, combined with the limitations inherent in the design of Mendelian randomization studies, it is challenging for us to make such a conversion. Moreover, the calculation of absolute risk is significantly influenced by the specific background risk of the population, and improper conversion may not accurately reflect the actual risk. Therefore, although understanding the translation of relative risk into absolute risk has potential value for clinical decision-making, our study focuses on exploring the potential causal relationship between the oral microbiome and breast cancer risk, without directly assessing absolute risk. This is determined by the aims, design, and data limitations of our study |
|  | d) | Consider plots to visualize results (e.g. forest plot, scatterplot of associations between genetic variants and outcome versus between genetic variants and exposure) |  | Forest plots are presented in Figure 2 and Figure 3, while scatter plots are provided in the Supplementary Figures. |
| 12 | **Assessment of assumptions** |  |  |  |
|  | a) | Report the assessment of the validity of the assumptions |  | The article reports the results of the validity assessment of the related hypotheses in multiple sections throughout the main text and appendices. Specifically, Supplementary Tables 1 and 2 detail the statistical power of the instrumental variables used in each association, presented as F-statistic values. Additionally, the Q-statistic is utilized to detect heterogeneity in the statistical models to assess their stability. |
|  | b) | Report any additional statistics (e.g., assessments of heterogeneity across genetic variants, such as *I^2^*, Q statistic or E-value) |  | Supplementary Tables 1 and 2 detail the statistical power of the instrumental variables used in each association, presented as F-statistic values. Additionally, the Q-statistic is utilized to detect heterogeneity in the statistical models to assess their stability |
| 13 | **Sensitivity analyses and additional analyses** |  |  |  |
|  | a) | Report any sensitivity analyses to assess the robustness of the main results to violations of the assumptions | 3 | This article employs five MR methods (IVW, Simple Mode, Weighted Mode, the weighted median (WM) method and the MR-Egger. If the results from multiple methods are consistent, it suggests that the causal relationship between the exposure and outcome identified in the analysis is more reliable. |
|  | b) | Report results from other sensitivity analyses or additional analyses | 3 | MR-Egger, MR-PRESSO and leave-one-out methods. |
|  | c) | Report any assessment of direction of causal relationship (e.g., bidirectional MR) |  | None. |
|  | d) | When relevant, report and compare with estimates from non-MR analyses | 9 | In the discussion section, we detailedly compared the results of MR with those from observational studies. |
|  | e) | Consider additional plots to visualize results (e.g., leave-one-out analyses) |  | Figures 3-4 and the supplementary figures. |
|  | **DISCUSSION** |  |  |  |
| 14 | **Key results** | Summarize key results with reference to study objectives | 7 | Our research is a pioneering effort to employ MR to scrutinize the causal association between the oral microbiome and BC. We executed stringent quality control procedures to counteract potential confounding factors and reverse causation in the selection of SNPs. The MR analysis identified significant associations between 30 species of tongue and 37 species of saliva bacteria and the incidence of BC. Intersecting the tongue and saliva bacterial species findings led us to identify six oral bacterial genera spread across five families. Our research has unveiled significant causal links between the microbiomes found in saliva and on the dorsal tongue and BC through the application of MR analysis. These connections were further substantiated using sequencing data derived from our case-control study. |
| 15 | **Limitations** | Discuss limitations of the study, taking into account the validity of the IV assumptions, other sources of potential bias, and imprecision. Discuss both direction and magnitude of any potential bias and any efforts to address them | 9 | The discussion section of the article extensively addresses the study's limitations through a detailed examination of the data sources, the three core assumptions, and the analytical process. This comprehensive approach ensures a thorough understanding of the context and potential constraints of the research findings. |
| 16 | **Interpretation** |  |  |  |
|  | a) | Meaning: Give a cautious overall interpretation of results in the context of their limitations and in comparison with other studies | 9 | The article devotes the largest section of its discussion to this topic, providing a reasoned interpretation of the MR results by comparing them with multiple published studies. This approach not only contextualizes the findings within the broader research landscape but also offers insights into how the study's results align with or diverge from existing knowledge. By engaging in comparative analysis, the discussion enriches the understanding of the study's contributions and limitations within the field of genetic epidemiology and Mendelian Randomization research. |
|  | b) | Mechanism: Discuss underlying biological mechanisms that could drive a potential causal relationship between the investigated exposure and the outcome, and whether the gene-environment equivalence assumption is reasonable. Use causal language carefully, clarifying that IV estimates may provide causal effects only under certain assumptions | 9 | The discussion section of the article elaborates on the detailed biological mechanisms through which oral microbiota may lead to breast cancer, such as systemic inflammation, modulation of immune response, or the production of carcinogenic metabolites. |
|  | c) | Clinical relevance: Discuss whether the results have clinical or public policy relevance, and to what extent they inform effect sizes of possible interventions | 9 | The discussion section of the article provides a detailed description of the clinical significance of researching the correlation between oral microbiota and breast cancer, such as its impact on the efficacy of breast cancer treatment and the potential of probiotic therapy for breast cancer. |
| 17 | **Generalizability** | Discuss the generalizability of the study results (a) to other populations, (b) across other exposure periods/timings, and (c) across other levels of exposure | 9 | This limitation underscores the need for caution when extrapolating these findings to a broader population. The study's focus on East Asian populations constrains the external validity of its conclusions. |
|  | **OTHER INFORMATION** |  |  |  |
| 18 | **Funding** | Describe sources of funding and the role of funders in the present study and, if applicable, sources of funding for the databases and original study or studies on which the present study is based | 10 | No fundings. |
| 19 | **Data and data sharing** | Provide the data used to perform all analyses or report where and how the data can be accessed, and reference these sources in the article. Provide the statistical code needed to reproduce the results in the article, or report whether the code is publicly accessible and if so, where | 10 | The data sets employed in the present investigation are accessible at the following website: https://db.cngb.org/search/project/CNP0001664. The original contributions presented in the 16S sequencing are publicly available. This data can be found here: https://www.ncbi.nlm.nih.gov/sra/PRJNA1036382. |
| 20 | **Conflicts of Interest** | All authors should declare all potential conflicts of interest | 9 | The authors declare that the research was conducted in the absence of any commercial or financial relationships that could be construed as a potential conflict of interest. |

This checklist is copyrighted by the Equator Network under the Creative Commons Attribution 3.0 Unported (CC BY 3.0) license.

1. Skrivankova VW, Richmond RC, Woolf BAR, Yarmolinsky J, Davies NM, Swanson SA, et al. Strengthening the Reporting of Observational Studies in Epidemiology using Mendelian Randomization (STROBE-MR) Statement. JAMA. 2021;under review.

2. Skrivankova VW, Richmond RC, Woolf BAR, Davies NM, Swanson SA, VanderWeele TJ, et al. Strengthening the Reporting of Observational Studies in Epidemiology using Mendelian Randomisation (STROBE-MR): Explanation and Elaboration. BMJ. 2021;375:n2233.
